# Associations Between Selected Xenobiotics and Antinuclear Antibodies in the National Health and Nutrition Examination Survey, 1999–2004

**DOI:** 10.1289/ehp.1409345

**Published:** 2015-08-07

**Authors:** Gregg E. Dinse, Todd A. Jusko, Irene Z. Whitt, Caroll A. Co, Christine G. Parks, Minoru Satoh, Edward K.L. Chan, Kathryn M. Rose, Nigel J. Walker, Linda S. Birnbaum, Darryl C. Zeldin, Clarice R. Weinberg, Frederick W. Miller

**Affiliations:** 1 Biostatistics Center, Social & Scientific Systems, Inc., Durham, North Carolina, USA; 2 Departments of Public Health Sciences and Environmental Medicine, University of Rochester School of Medicine and Dentistry, Rochester, New York, USA; 3 Division of Rheumatology and Immunology, Department of Internal Medicine, Duke University, Durham, North Carolina, USA; 4 Epidemiology Branch, National Institute of Environmental Health Sciences (NIEHS), National Institutes of Health (NIH), Department of Health and Human Services (DHHS), Research Triangle Park, North Carolina, USA; 5 Department of Clinical Nursing, University of Occupational and Environmental Health, Kitakyushu, Fukuoka, Japan; 6 Department of Oral Biology, University of Florida, Gainesville, Florida, USA; 7 Division of the National Toxicology Program, NIEHS, NIH, DHHS, Research Triangle Park, North Carolina, USA; 8 Laboratory of Toxicology and Toxicokinetics, National Cancer Institute, NIH, DHHS, Research Triangle Park, North Carolina, USA; 9 Division of Intramural Research, and; 10 Biostatistics and Computational Biology Branch, NIEHS, NIH, DHHS, Research Triangle Park, North Carolina, USA; 11 Environmental Autoimmunity Group, NIEHS, NIH, DHHS, Bethesda, Maryland, USA

## Abstract

**Background:**

Potential associations between background environmental chemical exposures and autoimmunity are understudied.

**Objectives:**

Our exploratory study investigated exposure to individual environmental chemicals and selected mixtures in relation to the presence of antinuclear antibodies (ANA), a widely used biomarker of autoimmunity, in a representative sample of the U.S. population.

**Methods:**

This cross-sectional analysis used data on 4,340 participants from the National Health and Nutrition Examination Survey (1999–2004), of whom 14% were ANA positive, to explore associations between ANA and concentrations of dioxins, dibenzofurans, polychlorinated biphenyls, organochlorines, organophosphates, phenols, metals, and other environmental exposures and metabolites measured in participants’ serum, whole blood, or urine. For dioxin-like compounds with toxic equivalency factors, we developed and applied a new statistical approach to study selected mixtures. Lognormal models and censored-data methods produced estimates of chemical associations with ANA in males, nulliparous females, and parous females; these estimates were adjusted for confounders and accommodated concentrations below detectable levels.

**Results:**

Several associations between chemical concentration and ANA positivity were observed, but only the association in males exposed to triclosan remained statistically significant after correcting for multiple comparisons (mean concentration ratio = 2.8; 95% CI: 1.8, 4.5; p < 0.00001).

**Conclusions:**

These data suggest that background levels of most xenobiotic exposures typical in the U.S. population are not strongly associated with ANA. Future studies should ideally reduce exposure misclassification by including prospective measurement of the chemicals of concern and should track changes in ANA and other autoantibodies over time.

**Citation:**

Dinse GE, Jusko TA, Whitt IZ, Co CA, Parks CG, Satoh M, Chan EKL, Rose KM, Walker NJ, Birnbaum LS, Zeldin DC, Weinberg CR, Miller FW. 2016. Associations between selected xenobiotics and antinuclear antibodies in the National Health and Nutrition Examination Survey, 1999–2004. Environ Health Perspect 124:426–436; http://dx.doi.org/10.1289/ehp.1409345

## Introduction

Autoimmune diseases are characterized by pathologic inflammation and autoantibodies or self-directed T lymphocyte responses. These acquired, often incurable, disorders affect up to 8% of the U.S. population, and many are rapidly increasing in prevalence for reasons that are unclear [Bach 2002; Jacobson et al. 1997; National Institutes of Health (NIH) 2005]. These diseases are major causes of death and disability among young and middle-aged women and have an enormous public health impact in the United States and worldwide (NIH 2005). Little is known about the causes of autoimmune diseases and the autoantibodies associated with them, but both genetic and environmental factors are likely to be involved (Ellis et al. 2014).

Although animal and human studies provide evidence of immunosuppression in relation to certain early- and later-life chemical exposures (e.g., low vaccine responses, thymic atrophy), autoimmune responses are less well-studied (Heilmann et al. 2010; Jusko et al. 2012; Lawrence and Kerkvliet 2006; Looker et al. 2014). However, a few studies indicate that some environmental factors, including drugs, tobacco smoke, silica, and various chemicals, are associated with autoimmune diseases and other immune effects (Miller 2011). Specific examples include polychlorinated biphenyls (PCBs) (Langer et al. 2008), hexachlorobenzene (Daniel et al. 2001; Loose et al. 1978; Michielsen et al. 1999; Queiroz et al. 1998a, 1998b; Schielen et al. 1993), and mercury (Bagenstose et al. 1999; Pollard et al. 2001; Via et al. 2003).

Among the most commonly measured biomarkers of autoimmunity are antinuclear antibodies (ANA), which are traditionally assessed by indirect immunofluorescence and are a heterogeneous group of autoantibodies targeting both nuclear and cytoplasmic components of cells (Tan 1989). Although ANA are associated with a number of autoimmune diseases, they can also develop in apparently healthy individuals after infections or following the use of medications; furthermore, their prevalence tends to be higher in parous females and the elderly (Hollingsworth et al. 1996; Parks et al. 2014; Satoh et al. 2007, 2012). Many persistent organic pollutants (POPs) exhibit hormone-disruption properties that could lead to increased ANA, and exposure to POPs has been hypothesized to increase the risk of systemic lupus erythematosus (Cooper et al. 1998). Indeed, some research has evaluated the prevalence of ANA in relation to 1-chloro-4-[2,2-dichloro-1-(4-chlorophenyl)ethenyl]benzene (Cooper et al. 2004), PCBs (Gallagher et al. 2013), asbestos (Pfau et al. 2005), and mercury (Bernhoft 2012; Gallagher and Meliker 2012; Lubick 2010; Nyland et al. 2011; Somers et al. 2015). However, to date, few studies have considered a broad range of background chemical exposures in relation to ANA.

In light of the limited information available about the effects of xenobiotics on autoimmunity, and given the availability of both ANA and chemical data for a large number of individuals in the National Health and Nutrition Examination Survey (NHANES), we assessed ANA associations with selected xenobiotics and mixtures by evaluating NHANES data from 1999 to 2004.

## Methods

*Study participants.* The NHANES data were collected by the U.S. Centers for Disease Control and Prevention (CDC) in 2-year cycles; we analyzed data from 1999–2000, 2001–2002, and 2003–2004 (http://www.cdc.gov/nchs/nhanes/nhanes_questionnaires.htm). From these cycles, NHANES staff used a multistage strategy to select a representative sample of 7,106 participants ≥ 12 years old for a substudy to assess serum levels of organochlorines. Of these, 4,754 had both chemical and ANA samples available for analysis. We excluded pregnant women and participants who self-reported as “other non-Hispanic race” (including non-Hispanic multiracial), reducing our sample size to 4,340. The NHANES data set provided extensive self-reported sociodemographic information and other health-related data. Constructed variables such as body mass index (BMI) and poverty index ratio (PIR) were also included (Lohman et al. 1988). We found no appreciable differences in demographic profiles between the larger substudy and our study sample (data not shown). The NHANES protocol was approved by the NCHS Research Ethics Review Board, and written informed consent was obtained from all participants (http://www.cdc.gov/nchs/nhanes/irba98.htm).

*Determination of ANA status.* ANA were measured in serum specimens with a standard immunofluorescence assay using commercial HEp-2 ANA slides (Inova Diagnostics) with 1:80 dilutions of sera (Satoh et al. 2012) and staining with DyLight 488-conjugated donkey antihuman IgG antibodies (Jackson ImmunoResearch) (Jakymiw et al. 2006). Staining intensities were graded from 0 to 4 relative to a standard reference gallery (http://www.cdc.gov/nchs/nhanes/nhanes1999-2000/SSANA_A.htm); intensities of 3 and 4 were defined as positive based on findings from commercial ANA reference laboratories (Chan et al. 2007; Satoh et al. 2012).

*Chemical measurements.* Given the exploratory nature of this study, we analyzed a diverse set of both persistent and nonpersistent chemicals. These included broad classes of compounds such as dioxins, dibenzofurans, PCBs, and other organochlorines, as well as metals, phenols, chloroacetanilides, organophosphates, pyrethroids, carbamate metabolites, cotinine, and other compounds and metabolites (Tables 1 and 2). The exception to this exploratory approach was compounds with dioxin-like activity, which have well-documented immunotoxic effects in animal studies (Lawrence and Vorderstrasse 2004). We decided *a priori* to include any chemical with a toxic equivalency factor (TEF) from the World Health Organization (WHO) (Van den Berg et al. 2006) (Table 1); other chemicals evaluated in the present study are listed in Table 2.

**Table 1:** Available data for dioxin-like chemicals for 4,340 participants studied in the 1999–2004 National Health and Nutrition Examination Surveys (NHANES).

Chemical (pg/g serum lipid)	TEF^*a*^	Number of observations (percent < LOD)^*b*^		
		Cycle 1: 1999–2000	Cycle 2: 2001–2002	Cycle 3: 2003–2004
Chlorinated dibenzo-*p*-dioxins
2,3,7,8-TCDD	1.00000	1,565 (100)	1,092 (87)	1,683 (63)
1,2,3,7,8-PnCDD	1.00000	1,554 (87)	1,087 (64)	1,683 (47)
1,2,3,4,7,8-HxCDD	0.10000	0	1,090 (65)	1,665 (75)
1,2,3,6,7,8-HxCDD	0.10000	1,523 (61)	1,086 (6)	1,673 (19)
1,2,3,7,8,9-HxCDD	0.10000	1,514 (87)	1,088 (58)	1,672 (73)
1,2,3,4,6,7,8-HpCDD	0.01000	1,519 (42)	1,070 (1)	1,677 (3)
1,2,3,4,6,7,8,9-OCDD	0.00030	1,544 (40)	1,033 (18)	1,656 (16)
Chlorinated dibenzofurans
2,3,4,7,8-PnCDF	0.30000	1,546 (62)	1,081 (33)	1,675 (35)
2,3,7,8-TCDF^*c*^	0.10000	1,546 (100)	1,084 (99)	1,673 (97)
1,2,3,4,7,8-HxCDF	0.10000	1,530 (64)	1,078 (17)	1,670 (40)
1,2,3,6,7,8-HxCDF	0.10000	1,538 (80)	1,089 (28)	1,671 (51)
1,2,3,7,8,9-HxCDF^*c*^	0.10000	1,519 (100)	1,078 (100)	1,668 (100)
2,3,4,6,7,8-HxCDF^*c*^	0.10000	1,527 (98)	1,083 (89)	1,669 (95)
1,2,3,7,8-PnCDF^*c*^	0.03000	1,559 (100)	1,085 (99)	1,671 (98)
1,2,3,4,6,7,8-HpCDF	0.01000	1,372 (57)	1,071 (10)	1,661 (10)
1,2,3,4,7,8,9-HpCDF^*c*^	0.01000	0	1,073 (100)	1,656 (94)
1,2,3,4,6,7,8,9-OCDF	0.00030	1,516 (99)	1,058 (100)	1,654 (73)
Dioxin-like polychlorinated biphenyls
3,3’,4,4’,5-Pentachlorobiphenyl (PCB126)	0.10000	1,544 (51)	1,079 (11)	1,664 (7)
3,3’,4,4’,5,5’-Hexachlorobiphenyl (PCB169)	0.03000	1,526 (53)	1,076 (11)	1,668 (42)
3,4,4’,5-Tetrachlorobiphenyl (PCB81)	0.00030	1,528 (99)	1,070 (100)	1,664 (64)
2,3,3’,4,4’-Pentachlorobiphenyl (PCB105)	0.00003	1,510 (89)	1,092 (76)	1,637 (3)
2,3’,4,4’,5-Pentachlorobiphenyl (PCB118)	0.00003	1,520 (60)	1,092 (24)	1,642 (0)
2,3,3’,4,4’,5-Hexachlorobiphenyl (PCB156)	0.00003	1,501 (71)	1,087 (40)	1,645 (18)
2,3,3’,4,4’,5’-Hexachlorobiphenyl (PCB157)	0.00003	1,497 (97)	1,086 (90)	1,631 (36)
2,3’,4,4’,5,5’-Hexachlorobiphenyl (PCB167)	0.00003	1,504 (95)	1,085 (87)	1,636 (42)
2,3,3’,4,4’,5,5’-Heptachlorobiphenyl (PCB189)	0.00003	0	1,090 (100)	1,596 (76)
A lone zero in the 1999–2000 column indicates that the chemical in that row was excluded from the mixtures analyses owing to missing data in at least one cycle (i.e., Cycle 1). Abbreviations: ANA, antinuclear antibodies; HpCDD, heptachlorodibenzo-*p*-dioxin; HpCDF, heptachlorodibenzofuran; HxCDD, hexachlorodibenzo-*p*-dioxin; HxCDF, hexachlorodibenzofuran; LOD, limit of detection; OCDD, octachlorodibenzo-*p*-dioxin; OCDF, octachlorodibenzofuran; PCB, polychlorinated biphenyl; PnCDD, pentachlorodibenzo-*p*-dioxin; PnCDF, pentachlorodibenzofuran; TCDD, tetrachlorodibenzo-*p*-dioxin; TCDF, tetrachlorodibenzofuran; TEF, toxic equivalency factor. ^***a***^The TEF values are the 2005 World Health Organization estimates (Van den Berg et al. 2006). ^***b***^The percent below the LOD can vary over time because it is a function of the concentration distribution, the volume of sample available for analysis, and the analytic method used to evaluate the sample. ^***c***^For survey years 1999–2004 combined, the overall percent below the LOD was ≥ 90%.				

**Table 2:** Available data for chemicals without a toxic equivalency factor for 4,340 participants studied in the 1999–2004 National Health and Nutrition Examination Surveys (NHANES).

Chemical or metabolite [units]	Matrix	Number of observations (percent < LOD)^*a*^		
		Cycle 1: 1999–2000	Cycle 2: 2001–2002	Cycle 3: 2003–2004
Non–dioxin-like polychlorinated biphenyls [ng/g]
2,4,4’-Trichlorobiphenyl (PCB28)	S	1,458 (98)	0	1,642 (0)
2,2’,3,5’-Tetrachlorobiphenyl (PCB44)	S	0	0	1,645 (0)
2,2’,4,5’-Tetrachlorobiphenyl (PCB49)	S	0	0	1,632 (1)
2,2’,5,5’-Tetrachlorobiphenyl (PCB52)	S	1,506 (99)	892 (90)	1,652 (0)
2,3’,4,4’-Tetrachlorobiphenyl (PCB66)	S	1,523 (97)	1,078 (89)	1,653 (1)
2,4,4’,5-Tetrachlorobiphenyl (PCB74)	S	1,515 (62)	1,092 (28)	1,653 (0)
2,2’,3,4,5’-Pentachlorobiphenyl (PCB87)	S	0	1,085 (99)	1,653 (17)
2,2’,4,4’,5-Pentachlorobiphenyl (PCB99)	S	1,493 (70)	1,077 (34)	1,632 (0)
2,2’,4,5,5’-Pentachlorobiphenyl (PCB101)	S	1,522 (99)	1,092 (96)	1,653 (3)
2,3,3’,4’,6-Pentachlorobiphenyl (PCB110)	S	0	1,085 (99)	1,639 (2)
2,2’,3,3’,4,4’-Hexachlorobiphenyl (PCB128)^*b*^	S	1,526 (99)	1,085 (100)	1,651 (76)
2,2’,3,4,4’,5’-Hexachlorobiphenyl (PCB138+158)	S	1,521 (65)	1,089 (5)	1,651 (0)
2,2’,3,4’,5,5’-Hexachlorobiphenyl (PCB146)	S	1,514 (76)	1,087 (48)	1,651 (2)
2,2’,3,4’,5’,6-Hexachlorobiphenyl (PCB149)	S	0	1,092 (100)	1,631 (5)
2,2’,3,5,5’,6-Hexachlorobiphenyl (PCB151)	S	0	1,092 (99)	1,632 (22)
2,2’,4,4’,5,5’-Hexachlorobiphenyl (PCB153)	S	1,518 (60)	1,092 (3)	1,651 (0)
2,2’,3,3’,4,4’,5-Heptachlorobiphenyl (PCB170)	S	1,422 (62)	1,089 (20)	1,648 (3)
2,2’,3,3’,4,5,5’-Heptachlorobiphenyl (PCB172)	S	1,499 (96)	1,066 (81)	1,647 (36)
2,2’,3,3’,4,5’,6’-Heptachlorobiphenyl (PCB177)	S	1,482 (93)	1,078 (80)	1,645 (20)
2,2’,3,3’,5,5’,6-Heptachlorobiphenyl (PCB178)	S	1,523 (91)	1,087 (77)	1,651 (25)
2,2’,3,4,4’,5,5’-Heptachlorobiphenyl (PCB180)	S	1,517 (56)	1,090 (9)	1,652 (1)
2,2’,3,4,4’,5’,6-Heptachlorobiphenyl (PCB183)	S	1,522 (86)	1,092 (65)	1,648 (12)
2,2’,3,4’,5,5’,6-Heptachlorobiphenyl (PCB187)	S	1,520 (61)	1,092 (29)	1,644 (2)
2,2’,3,3’,4,4’,5,5’-Octachlorobiphenyl (PCB194)	S	0	1,083 (33)	1,607 (22)
2,2’,3,3’,4,4’,5,6-Octachlorobiphenyl (PCB195)	S	0	1,072 (100)	1,601 (46)
2,2’,3,3’,4,4’,5,6’-Octachlorobiphenyl (PCB196+203)	S	0	1,088 (39)	1,642 (14)
2,2’,3,3’,4,5,5’,6’-Octachlorobiphenyl (PCB199)	S	0	1,083 (36)	1,627 (14)
2,2’,3,3’,4,4’,5,5’,6-Nonachlorobiphenyl (PCB206)	S	0	1,050 (86)	1,631 (7)
Decachlorobiphenyl (PCB209)	S	0	0	1,618 (7)
Organochlorines
1-Chloro-2-[2,2,2-trichloro-1-(4-chlorophenyl)ethyl]benzene (o,p´-DDT) [pg/g]^*b*^	S	1,323 (99)	1,076 (99)	0
1-Chloro-4-[2,2,2-trichloro-1-(4-chlorophenyl)ethyl]benzene (p,p´-DDT) [pg/g]	S	1,332 (70)	1,092 (60)	0
1-Chloro-4-[2,2-dichloro-1-(4-chlorophenyl)ethenyl]benzene (p,p´-DDE) [pg/g]	S	1,549 (0)	1,090 (0)	0
Hexachlorobenzene [pg/g]^*b*^	S	1,345 (98)	1,077 (91)	0
2,4,5-Trichlorophenol (2,4,5-TCP) [μg/g]	U	0	0	1,648 (64)
2,4,6-Trichlorophenol [μg/g]	U	1,045 (16)	1,053 (49)	1,648 (68)
Pentachlorophenol [μg/g]	U	0	0	1,536 (64)
Aldrin [ng/g]^*b*^	S	0	1,070 (100)	0
*beta*-Hexachlorocyclohexane [ng/g]	S	1,501 (36)	1,077 (25)	0
Dieldrin [ng/g]	S	0	1,021 (32)	0
Endrin [ng/g]^*b*^	S	0	1,028 (100)	0
*gamma*-Hexachlorocyclohexane [ng/g]^*b*^	S	1,428 (97)	1,070 (99)	0
Heptachlor epoxide [ng/g]	S	1,265 (66)	1,065 (37)	0
Mirex [ng/g]^*c*^	S	1,451 (92)	1,078 (64)	0
Oxychlordane [ng/g]	S	1,321 (46)	1,057 (16)	0
*trans*-Nonachlor [ng/g]	S	1,527 (30)	1,075 (9)	0
Metals
Cadmium [μg/L]	WB	1,564 (23)	1,091 (26)	1,681 (23)
Lead [μg/dL]	WB	1,564 (0)	1,091 (0)	1,681 (0)
Mercury, total blood [μg/L]^*d*^	WB	369 (7)	276 (5)	1,681 (8)
Mercury, inorganic blood [μg/L]^*d*^	WB	369 (97)	272 (93)	1,656 (74)
Mercury, urinary [μg/g]^*e*^	U	358 (11)	266 (13)	0
Phenols [μg/g]
Bisphenol A (BPA)	U	0	0	1,648 (7)
Triclosan	U	0	0	1,648 (25)
Benzophenone-3	U	0	0	1,648 (3)
Chloroacetanilides [μg/g]
Acetochlor mercapturate^*b*^	U	0	1,055 (98)	0
Alachlor mercapturate^*c*^	U	1,026 (66)	0	0
Metolachlor mercapturate^*b*^	U	0	1,067 (97)	0
Organophosphates [μg/g]
Dimethylphosphate (DMP)	U	0	0	1,631 (49)
Diethylphosphate (DEP)	U	0	0	1,598 (47)
Dimethylthiophosphate (DMTP)	U	0	0	1,631 (20)
Diethylthiophosphate (DETP)	U	0	0	1,610 (48)
Dimethyldithiophosphate (DMDTP)	U	0	0	1,610 (58)
Diethyldithiophosphate (DEDTP)^*b*^	U	0	0	1,631 (91)
3-Chloro-7-hydroxy-4-methyl-2H-chromen-2-one/ol^*b*^	U	0	1,039 (97)	0
3,5,6-Trichloro-2-pyridinol	U	1,050 (7)	1,050 (28)	0
Diethylaminomethylpyrimidinol/one^*b*^	U	0	1,047 (95)	0
Malathion dicarboxylic acid	U	1,023 (46)	0	0
*para*-Nitrophenol	U	1,049 (76)	1,038 (51)	0
Oxypyrimidine	U	956 (68)	1,067 (96)	0
Pyrethroids [μg/g]
4-Fluoro-3-phenoxybenzoic acid^*b*^	U	1,024 (96)	1,068 (100)	0
*cis*-3-(2,2-Dibromovinyl)-2,2-dimethylcyclopropane carboxylic acid^*b*^	U	895 (100)	1,068 (99)	0
*cis*-3-(2,2-Dichlorovinyl)-2,2-dimethylcyclopropane carboxylic acid (cis-Cl2CA)	U	1,029 (56)	1,068 (66)	0
3-Phenoxybenzoic acid	U	1,052 (29)	1,068 (25)	0
*trans*-3-(2,2-Dichlorovinyl)-2,2-dimethylcyclopropane carboxylic acid (trans-Cl2CA)	U	1,042 (66)	1,063 (75)	0
Carbamates [μg/g]
2-Isopropoxyphenol^*b*^	U	1,007 (97)	1,053 (100)	1,556 (100)
Carbofuranphenol^*b*^	U	1,049 (87)	1,061 (100)	1,557 (100)
Tobacco smoke exposure [ng/mL]
Cotinine	S	1,548 (37)	1,085 (25)	1,681 (16)
Other compounds [μg/g]
Atrazine mercapturate^*b*^	U	1,000 (95)	1,042 (99)	0
2,4-Dichlorophenol	U	0	0	1,648 (16)
*N,N*-Diethyl-3-methylbenzamide (DEET)	U	1,036 (84)	1,067 (88)	0
*ortho*-Phenylphenol	U	0	0	1,648 (45)
2,5-Dichlorophenol	U	0	0	1,648 (1)
2,4-Dichlorophenoxyacetic acid	U	1,041 (46)	1,022 (74)	0
2,4,5-Trichlorophenoxyacetic acid^*b*^	U	969 (96)	1,067 (100)	0
Abbreviations: ANA, antinuclear antibodies; LOD, limit of detection; PCB, polychlorinated biphenyl; S, serum; U, urine; WB, whole blood. ^***a***^The percent below the LOD can vary over time because it is a function of the concentration distribution, the volume of sample available for analysis, and the analytic method used to evaluate the sample. ^***b***^For survey years 1999–2004 combined, the overall proportion below the LOD was ≥ 90%. ^***c***^< 6 nulliparous female participants were ANA positive and had a detectable concentration. ^***d***^No data were available for males in survey years 1999–2002. ^***e***^No data were available for females in survey years 2003–2004 or for males in any survey years.				

Although we aimed to be as broad as possible in our assessment of exposures, there were several complicating factors. First, some chemicals were undetectable in nearly all participant samples, with concentrations below the assay’s limit of detection (LOD) (Browne and Whitcomb 2010). Although we used statistical methods developed to handle large proportions of nondetects (Dinse et al. 2014; Helsel 2012), we excluded chemicals for which the overall proportion (across all cycles) of undetectable concentrations exceeded 90% [e.g., 1,2,3,7,8-pentachlorodibenzofuran (PnCDF); Table 1] because statistical estimates could become unstable in these cases. Second, measured concentrations of some compounds of interest, such as perfluoroalkyl substances, were not determined in the NHANES participants with ANA data, further limiting the number of environmental chemicals available for the present study.

Chemicals or their metabolites were measured in the serum, whole blood, or urine of NHANES participants. All specimens were analyzed by the Division of Laboratory Sciences, National Center for Environmental Health, Atlanta, Georgia (CDC 2005, 2009). For quantitative summaries of exposure levels, see the tables in CDC (2009) and Crinnion (2010). In addition, quantitative summaries of LOD values are given in Appendix D of CDC (2009).

*Individual chemicals and dioxin-like mixtures.* We investigated various individual chemicals, as well as several mixtures of dioxin-like chemicals that have TEFs. Three mixture groupings (chlorinated dibenzo-*p*-dioxins, chlorinated dibenzofurans, and dioxin-like PCBs) and the TEFs of their component chemicals are shown in Table 1. When assessing these mixtures, TEFs are used as adjustment factors to transform component concentrations to a common potency scale relative to 2,3,7,8-tetrachlorodibenzo-*p*-dioxin (TCDD). Each TEF is based on expert judgment of the relative potency of a given dioxin-like chemical to that of TCDD, derived predominantly from *in vivo* rodent experiments that assessed responses induced by the aryl hydrocarbon (Ah) receptor (Van den Berg et al. 2006). Once the component concentrations have been expressed in equal potency units, they are summed to create a toxic equivalent (TEQ) concentration for the mixture.

*Selection of confounders.* Our previously reported analyses (Parks et al. 2014; Satoh et al. 2012) showed a greater prevalence of ANA in female versus male, parous versus nulliparous female, old versus young, normal weight versus overweight/obese, and non-Hispanic black versus non-Hispanic white. The present study confirms these associations (see Supplemental Material, Table S1) in addition to an association between ANA and time period. These factors are often predictors of chemical concentrations in NHANES (Chen et al. 2010; Ye et al. 2014) and were associated with many chemicals investigated in the present study; therefore, we considered them as possible confounders in our analyses. We also included poverty index ratio (PIR) because socioeconomic status is associated with autoimmune diseases (Calixto and Anaya 2014) and with many chemicals in our study.

*Statistical model.* Large proportions of nondetectable concentrations, which was the case for many chemicals in our study, can complicate the usual modeling of ANA positivity as a function of chemical concentration and various confounders. We addressed this problem by treating analyte concentration as the dependent variable and ANA status as a covariate (Dinse et al. 2014), incorporating nondetects as left-censored data and applying conventional survival methods that adjust for confounders and incorporate quantifiable analyte measurements. Our main analysis assumed a lognormal distribution for chemical concentration, a standard choice (Ott 1994) that implies log concentration is normally distributed, although we also performed parametric sensitivity analyses based on exponential, Weibull, gamma, and log-logistic distributions, and a semi-parametric sensitivity analysis based on a reverse-scale Cox method (Dinse et al. 2014). The mean log concentration was modeled by a linear function of covariates; thus, covariate effects on mean concentration were multiplicative (see Supplemental Material, “Statistical Model”). We assessed the association between chemical concentration and ANA via the sign, magnitude, and statistical significance of the estimated regression coefficient for ANA. A default alpha level of 0.05 was used to judge statistical significance. The regression models excluded participants with missing covariate values, reducing the sample size to 3,754 in the adjusted analyses.

The LIFEREG procedure in SAS (v9.3; SAS Institute, Cary, NC) was used to perform the lognormal regression analyses, where the outcome variable was either an individual chemical’s concentration or a mixture’s TEQ concentration. Concentrations of lipophilic compounds were modeled on a per-lipid basis, and those determined in urine were modeled on a creatinine basis to account for dilution. We compared the results on a per-lipid basis with results obtained when including total lipid concentration as a covariate instead of dividing analyte concentration by total lipid concentration (Schisterman et al. 2005), and we observed little difference (data not shown). Thus, we chose to model concentrations of lipophilic compounds on a per-lipid basis. We ran a similar sensitivity analysis for chemicals measured in urine, including creatinine as a covariate rather than dividing analyte concentration by creatinine concentration. The results were not materially different (data not shown); therefore, we chose to model those concentrations on a creatinine basis. Regarding the small differences cited in these sensitivity analyses, we examined each chemical’s regression coefficient for ANA and obtained similar estimates using both models; among the estimated coefficients that were statistically significantly different from zero, the signs were the same under both models, and the magnitudes were very close.

Our analyses included ANA status and potential confounders as covariates. To fully adjust for sex and parity, we performed separate analyses for males, nulliparous females, and parous females. Stratification on parity simplified the modeling and was based on evidence that nulliparous and parous women differ in ANA prevalence and possibly in how ANA relates to other factors such as age (Parks et al. 2014). The potential confounders considered were race/ethnicity, time period, BMI, age, and PIR. We used categorical variables to summarize race/ethnicity (non-Hispanic white, non-Hispanic black, Hispanic), time period (1999–2000, 2001–2002, 2003–2004), and BMI (underweight, normal, overweight, obese). We treated age and PIR as quantitative (continuous) variables, using a restricted cubic spline (Harrell 2001) for age and a linear term for PIR. Allowing confounder categories to act as ANA effect modifiers generally did not provide a statistically significant improvement in model fit, so we did not include ANA-by-confounder interactions in our primary analyses. However, as a post hoc analysis to further investigate the association between ANA and one particular chemical (triclosan), we fitted several expanded models, with each adding a two-way interaction between ANA and a given confounder.

Appropriate statistical interpretations depend on having adequate data. Thus, within each sex/parity group, we excluded any chemical for which fewer than six ANA-positive participants had a detectable concentration. This procedure eliminated one chemical in males (urinary mercury) and two in nulliparous females (mirex and alachlor mercapturate).

*Assessing chemical–ANA associations.* Associations between chemical concentration and ANA were estimated using the ANA regression coefficient. Because ANA effects in nulliparous and parous females were often similar, we simplified the reporting of some results by calculating combined estimates for all females as weighted averages of parity-specific estimates using inverse variance estimates as weights. However, rather than work directly with the ANA regression coefficient, we exponentiated it to obtain a parameter that was interpretable as the ratio of mean concentrations for ANA-positive versus ANA-negative participants (see Supplemental Material, “Statistical Model”). Estimates of this mean concentration ratio (MCR) > 1 corresponded to positive associations between chemical concentration and ANA (i.e., persons with higher concentrations had a higher prevalence of ANA). Similarly, an MCR < 1 corresponded to a negative chemical-ANA association, such that persons with higher concentrations had a lower prevalence of ANA. An MCR = 1 corresponded to no association between ANA and the chemical. Logarithmic distance from 1 reflects association strength.

*Accounting for censoring.* Nondetectable concentrations were left-censored, known only to be less than the LOD, and a mixture TEQ was interval-censored if some component concentrations were below the LOD and others were not, in which case the TEQ was known to be between a lower limit and an upper limit (see Supplemental Material, “Accounting for censoring”). If all information on a component chemical was missing, the TEQ censoring interval ranged from zero to infinity and was uninformative. Rather than exclude such persons, however, we calculated their TEQs by treating missing concentrations as censored in the interval from zero to the largest observed concentration for that chemical. As a sensitivity analysis, we compared the results obtained when excluding and including those with missing component concentrations; both analyses yielded similar results (data not shown).

*Accounting for sampling.* The NHANES data were obtained from a multistage stratified cluster sample. The LIFEREG procedure does not incorporate information on sampling strata and clusters; therefore, although it properly estimates regression coefficients, it does not account for the correlation structure when estimating variances. Thus, when constructing confidence intervals (CIs) for regression coefficients, we used a jackknife procedure to provide standard errors appropriate for complex survey data (see Supplemental Material, “Accounting for sampling”). We ignored probability sampling weights to improve efficiency for assessing chemical–ANA associations, exploiting the fact that our analysis conditions on variables that influenced the sampling (Korn and Graubard 1999).

*Accounting for multiple comparisons.* Because many chemicals were investigated, we used a Bonferroni correction to adjust statistical significance for multiple comparisons. We report both uncorrected and corrected results. Consistent with the exploratory nature of our study, uncorrected results with *p* < 0.05 can be used to generate hypotheses for future investigation, although many may later prove to be false positives. Bonferroni correction is fairly conservative; therefore, associations that remain statistically significant after adjustment are more likely to be true positives. We also applied the false discovery rate approach (Benjamini and Hochberg 1995) for comparison, which is less conservative than the Bonferroni method, and obtained similar results (data not shown).

## Results

*Participant descriptors.* Of the 4,340 NHANES participants in our analysis, 623 (14.4%) were ANA positive, which is consistent with previous ANA prevalence estimates of 13.8% (Satoh et al. 2012), 13.3% (Tan et al. 1997), and 12.9% (Mariz et al. 2011). In addition, of these 4,340 participants, 51% were males, 29% were parous females, 17% were nulliparous females, and 3% were females with no information on parity (see Supplemental Material, Table S1). The distribution of participants across categories of race/ethnicity, time period, age, PIR, and BMI, as well as category-specific ANA positivity percentages, are also shown in the Supplemental Material, Table S1. Multiple logistic regression produced odds ratios that confirmed an association between ANA and several of the covariates in our analysis (see Supplemental Material, Table S1).

*Dioxin-like chemicals.* We investigated 26 dioxin-like chemicals for which information was available in NHANES; these chemicals were classified into 7 chlorinated dibenzo-*p*-dioxins, 10 chlorinated dibenzofurans, and 9 dioxin-like PCBs (Table 1). We analyzed 21 of the chemicals individually after excluding 5 chlorinated dibenzofurans because > 90% of their concentrations were below the LOD. We also analyzed mixtures of chemicals within categories as well as an overall mixture of dioxin-like chemicals. The mixture analyses excluded 3 chemicals without data in one NHANES cycle, 1 of which had already been eliminated because of heavy censoring. Therefore, the mixture analyses involved 19 dioxin-like chemicals, comprising 6 chlorinated dibenzo-*p*-dioxins, 5 chlorinated dibenzofurans, and 8 dioxin-like PCBs.

**Figure 1:**
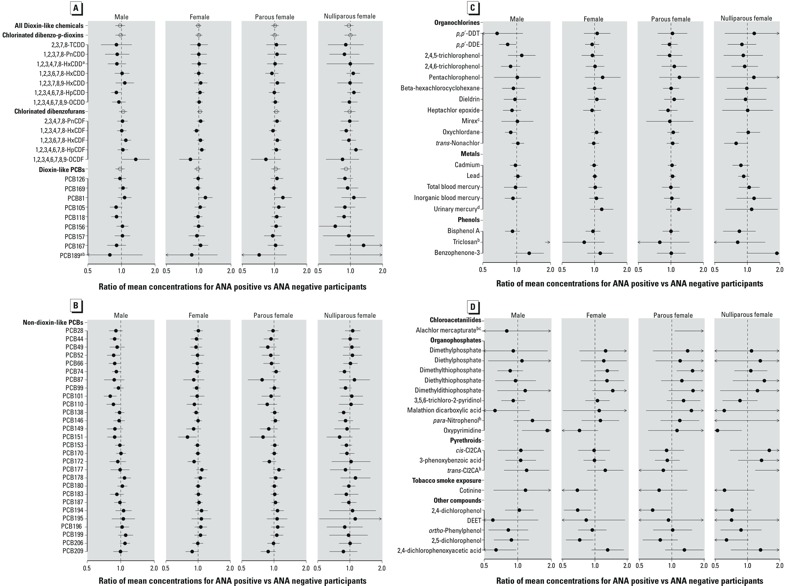
Estimated ANA positivity effects by sex and parity for individual chemicals and dioxin-like chemical mixtures, National Health and Nutrition Examination Survey, 1999–2004. Estimated ratios of mean concentrations (MCRs) for ANA-positive versus ANA-negative participants are plotted as solid dots for 21 dioxin-like chemicals in panel *A* and for 66 non–dioxin-like chemicals in panels *B–D*. Analogous estimates for dioxin-like chemical mixtures, both overall and within categories, are plotted as open circles in panel *A*. All estimates are adjusted for age, race/ethnicity, time period, BMI, and PIR. Values below (above) 1.0 indicate that those positive for ANA had a lower (higher) mean concentration of the chemical or mixture than those negative for ANA. The horizontal lines represent 95% confidence intervals, and left (right) arrowheads indicate that values extend below 0.5 (above 2.0). Results are shown separately by sex and parity, with overall female estimates calculated from inverse-variance weighted averages of parity-specific estimates. ***^a^***Two chemicals [1,2,3,4,7,8-hexachlorodibenzo-*p*-dioxin (HxCDD) and 2,3,3’,4,4’,5,5’-heptachlorobiphenyl (PCB189)] were excluded from mixture estimates because of missing data for 1999–2000. ***^b^***Five chemicals had MCRs below 0.5 or above 2.0 in one sex/parity group; therefore, no solid dot was plotted. The unplotted MCRs were 3.1 (95% CI: 0.6, 15.1) for PCB189 in nulliparous females, 2.8 (95% CI: 1.8, 4.5) for triclosan in males, 3.8 (95% CI: 1.1, 13.7) for alachlor mercapturate in parous females, 0.5 (95% CI: 0.1, 2.4) for *para*-ntirophenol in nulliparous females, and 2.1 (95% CI: 1.8, 3.9) for *trans*-3-(2,2-dichlorovinyl)-2,2-dimethylcyclopropanecarboxylic acid (trans-Cl2CA) in nulliparous females. ***^c^***For two chemicals (mirex and alachlor mercapturate), < 6 nulliparous females were ANA positive and had detectable concentrations; therefore, nothing was plotted for nulliparous females or for all females combined. ***^d^***One chemical (urinary mercury) had no data for males; therefore, nothing was plotted for males.

Overall, there was little evidence that ANA were associated with any of the dioxin-like chemicals or their mixtures. Only two dioxin-like chemicals were statistically significantly associated with ANA at the 0.05 level (Figure 1). Those chemicals were 1,2,3,4,6,7,8,9-octachlorodibenzo-*p*-dioxin (OCDF) in males (MCR = 1.3; 95% CI: 1.0, 1.8; *p* = 0.04) and 1,2,3,4,6,7,8-heptachlorodibenzo-*p*-dioxin (HpCDD) in males (MCR = 0.9; 95% CI: 0.8, 1.0; *p* = 0.05). Among all of the dioxin-like chemicals, the only one with an MCR larger than the 1.3 observed for 1,2,3,4,6,7,8,9-OCDF in males was PCB189 in nulliparous females (MCR = 3.1; 95% CI: 0.6, 15.1; *p* = 0.16), although this MCR was not significantly greater than 1. With regard to the mixture concentrations, neither the overall TEQ nor any category-specific TEQ was significantly associated with ANA regardless of sex or parity (Figure 1).

**Figure 2:**
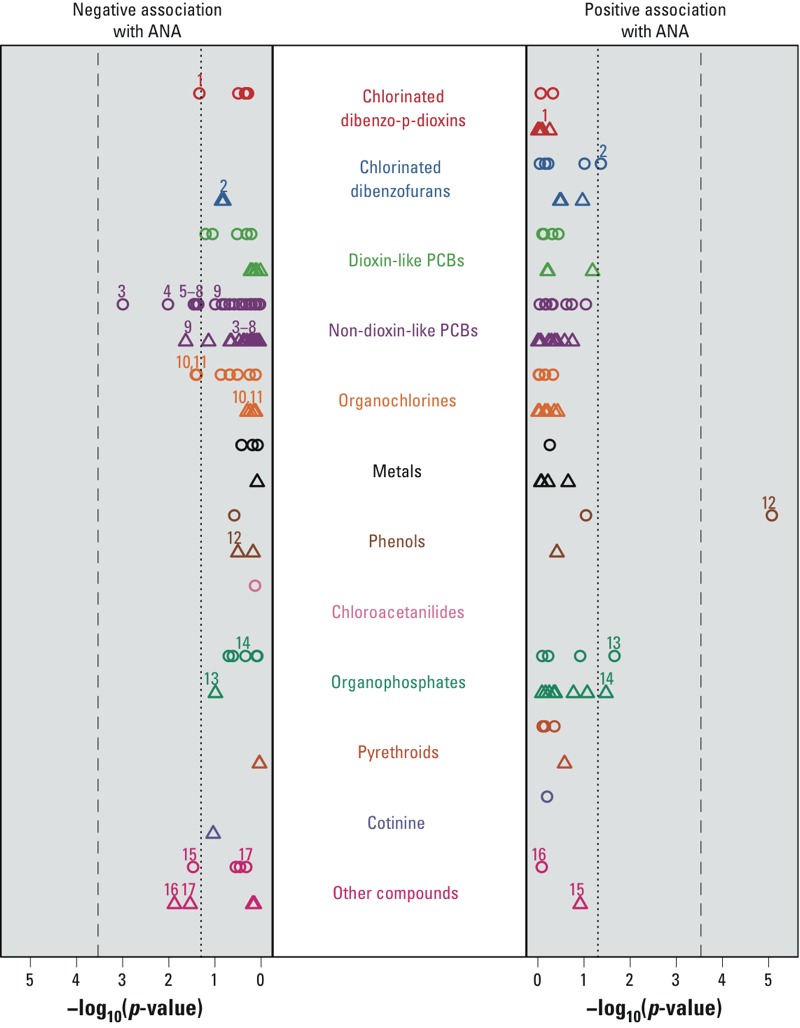
Statistical significance of associations between ANA and selected xenobiotics by sex, National Health and Nutrition Examination Survey, 1999–2004. For each chemical, the statistical significance of the ANA regression coefficient was calculated separately for males and females, under a lognormal concentration model adjusted for age, race/ethnicity, time period, BMI, and PIR. Chemicals are arranged within color-coded categories along the vertical axis, and negative log *p*-values are shown along the horizontal axes. Results are depicted by circles for males and triangles for females, where results for females were calculated from inverse-variance weighted averages of the parity-specific estimates. Symbols displayed on the right (left) indicate positive (negative) associations between ANA and the chemical. The dotted line corresponds to a *p*-value of 0.05 and the dashed line to the Bonferroni significance level, which is 0.05 divided by 171, the number of tests performed (86 for males and 85 for females). Chemicals significant at the uncorrected 0.05 level in at least one sex are labeled for both sexes. The chemical labels are: 1 = 1,2,3,4,6,7,8-heptachlorodibenzo-*p*-dioxin (HpCDD); 2 = 1,2,3,4,6,7,8,9-octachlorodibenzofuran (OCDF); 3 = 2,2',4,5,5'-pentachlorobiphenyl (PCB101); 4 = 2,2',3,5'-tetrachlorobiphenyl (PCB44); 5 = 2,3,3',4',6-pentachlorobiphenyl (PCB110); 6 = 2,2',5,5'-tetrachlorobiphenyl (PCB52); 7 = 2,3',4,4'-tetrachlorobiphenyl (PCB66); 8 = 2,4,4',5-tetrachlorobiphenyl (PCB74); 9 = 2,2’,3,5,5’,6-hexachlorobiphenyl (PCB151); 10 = 1-chloro-4-[2,2-dichloro-1-(4-chlorophenyl)ethenyl]benzene (*p,p*´-DDE); 11 = oxychlordane; 12 = triclosan; 13 = oxypyrimidine; 14 = dimethylthiophosphate; 15 = 2,4-dichlorophenoxyacetic acid; 16 = 2,4-dichlorophenol; 17 = 2,5-dichlorophenol.

We also summarized sex-specific associations between chemical concentration and ANA in terms of statistical significance (Figure 2). The two associations noted above (1,2,3,4,6,7,8,9-OCDF and 1,2,3,4,6,7,8-HpCDD, both in males) were statistically significant at the uncorrected 0.05 level, but not after correcting for multiple comparisons.

*Non–dioxin-like chemicals.* We investigated 83 non–dioxin-like chemicals, which were subdivided into 10 categories: 29 non–dioxin-like PCBs, 16 organochlorines, 5 metals, 3 phenols, 3 chloroacetanilides, 12 organophosphates, 5 pyrethroids, 2 carbamates, 1 biomarker of tobacco smoke exposure, and 7 other compounds (Table 2). Excluding chemicals with > 90% of their concentrations below the LOD left 66 non–dioxin-like chemicals in 9 categories: 28 PCBs, 11 organochlorines, 5 metals, 3 phenols, 1 chloroacetanilide, 9 organophosphates, 3 pyrethroids, 1 biomarker of tobacco smoke exposure, and 5 other compounds.

For each non–dioxin-like chemical, an estimate of the MCR and its 95% CI are shown in Figure 1 for each sex/parity group. Without correcting for multiple comparisons, 15 non–dioxin-like chemicals showed some evidence of an association with ANA (*p* < 0.05). Of these, 11 associations were in males: triclosan (MCR = 2.8; 95% CI: 1.8, 4.5; *p* < 0.00001), PCB101 (MCR = 0.8; 95% CI: 0.7, 0.9; *p* = 0.001), PCB44 (MCR = 0.9; 95% CI: 0.8, 1.0; *p* = 0.01), oxypyrimidine (MCR = 1.8; 95% CI: 1.1, 3.1; *p* = 0.02), PCB110 (MCR = 0.9; 95% CI: 0.7, 1.0; *p* = 0.03), 2,4-dichlorophenoxyacetic acid (MCR = 0.6; 95% CI: 0.4, 1.0; *p* = 0.03), PCB52 (MCR = 0.9; 95% CI: 0.8, 1.0; *p* = 0.04), 1-chloro-4-[2,2-dichloro-1-(4-chlorophenyl)ethenyl]benzene (*p,p*′-DDE) (MCR = 0.8; 95% CI: 0.7, 1.0; *p* = 0.04), PCB66 (MCR = 0.9; 95% CI: 0.8, 1.0; *p* = 0.04), PCB74 (MCR = 0.9; 95% CI: 0.8, 1.0; *p* = 0.04), and oxychlordane (MCR = 0.9; 95% CI: 0.8, 1.0; *p* = 0.04). There were 4 suggestive associations in females: 2,4-dichlorophenol (MCR = 0.7; 95% CI: 0.5, 0.9; *p* = 0.01), PCB151 (MCR = 0.8; 95% CI: 0.7, 1.0; *p* = 0.02), 2,5-dichlorophenol (MCR = 0.7; 95% CI: 0.5, 1.0; *p* = 0.03), and dimethylthiophosphate (MCR = 1.3; 95% CI: 1.0, 1.7; *p* = 0.03).

Not only does Figure 1 illustrate the sex-specific associations mentioned above, but it also shows the parity-specific associations in females. Although none of these associations was statistically significant after correcting for multiple testing, 4 associations were suggestive (*p* < 0.05) in nulliparous females: *trans*-3-(2,2-dichlorovinyl)-2,2-dimethylcyclopropane carboxylic acid (trans-Cl2CA) (MCR = 2.1; 95% CI: 1.2, 3.9; *p* = 0.01), oxypyrimidine (MCR = 0.5; 95% CI: 0.3, 0.9; *p* = 0.01), PCB138 (MCR = 0.9; 95% CI: 0.8, 1.0; *p* = 0.02), and PCB74 (MCR = 0.9; 95% CI: 0.8, 1.0; *p* = 0.05). There were also two suggestive associations in parous females, dimethylthiophosphate (MCR = 1.6; 95% CI: 1.1, 2.2; *p* = 0.01) and alachlor mercapturate (MCR = 3.8; 95% CI: 1.1, 13.7; *p* = 0.04); the former was also noted for all females combined (MCR = 1.3; 95% CI: 1.0, 1.7; *p* = 0.03).

The statistical significance of associations between ANA and non–dioxin-like chemicals was plotted separately for males and females (Figure 2). Of the 15 non–dioxin-like chemicals associated with ANA at the 0.05 level in either sex, only one association remained statistically significant after correcting for multiple comparisons: triclosan in males (MCR = 2.8; 95% CI: 1.8, 4.5; *p* < 0.00001), where creatinine-adjusted concentrations were higher in ANA-positive participants than in ANA-negative participants (see Supplemental Material, Figure S1). The nonparametric curves in Supplemental Material Figure S1 were constructed using the methods of Kaplan and Meier (1958) with concentrations below the LOD treated as left-censored observations; these curves were not adjusted for covariates.

Our primary regression model was adjusted for confounders but did not allow ANA effects to vary with confounders because in nearly all cases, the improvement in model fit due to adding interactions was not statistically significant. However, to further investigate the association between ANA and triclosan in males, we fitted several expanded models, with each adding a two-way interaction between ANA and a given confounder. The positive association between ANA and triclosan appeared to be subject to effect modification by age but not by race/ethnicity, BMI, or PIR. The MCR estimates were 2.6 (95% CI: 1.2, 5.4) in the 12–19 age group, 1.3 (95% CI: 0.6, 2.9) in the 20–54 age group, and 7.1 (95% CI: 3.5, 14.8) in the ≥ 55 age group (overall *p* = 0.03).

## Discussion

In general, our results did not suggest strong associations between the studied background xenobiotic exposures and ANA in this population-representative survey. These null results were consistent across classes of chemicals and across sex/parity groups. To our knowledge, this is the most comprehensive study to date of xenobiotic exposures and their possible associations with ANA.

Although our results for ANA were generally null, some chemicals showed weak associations that did not meet the Bonferroni level of significance but may warrant further consideration in future investigations because we cannot rule out their involvement in immune alterations that could lead to autoimmunity. The strong association between elevated triclosan concentrations and ANA positivity in males deserves comment. Triclosan is an antimicrobial used in a wide variety of consumer products such as toothpastes, soaps, and toys that works by blocking the active site of enoyl-acyl carrier protein reductase, an enzyme essential for fatty acid synthesis in bacteria (Fang et al. 2010; Yueh et al. 2014). The primary route of excretion of enoyl-acyl carrier protein reductase is via urination, and the estimated half-life of this enzyme is approximately 11 hr in urine. (Calafat et al. 2008; Fang et al. 2010; Sandborgh-Englund et al. 2006). Despite its short half-life, urinary measures of triclosan appear to be less variable over time than those of other phenols, such as bisphenol A (BPA) (Bertelsen et al. 2014; Koch et al. 2014; Meeker et al. 2013). Thus, the concentration of triclosan in a spot urine sample, such as those collected for NHANES, may serve as a reasonable biomarker of triclosan exposure. In terms of the potential immunotoxicity of triclosan, Clayton et al. (2011), using NHANES data, observed a positive association between urinary triclosan concentrations and the odds of having been diagnosed with allergies or hay fever, and others have also reported positive associations between urinary triclosan concentrations and allergic sensitization (Bertelsen et al. 2013). Similar results have been observed in female mice, where exposure to triclosan enhanced the hypersensitivity response to an allergen (Anderson et al. 2013). Although it is unclear how triclosan could be related to the development of autoimmunity, and why the association was only seen in males in our study, the enhancement of certain T-cell responses is thought to be strongly associated with the development of autoimmunity and autoimmune disease related to environmental exposures (Selmi et al. 2012). To address public health concerns, more studies are needed of populations exposed to high levels of triclosan; ideally, these studies should follow markers of immune function before, during, and after exposure.

A few small studies have reported associations between ANA positivity and various chemicals (Cebecauer et al. 2009; Cooper et al. 2004; Daniel et al. 2001; Kilburn and Warshaw 1992; Rosenberg et al. 1999), and some investigations of associations between exposures and ANA have been conducted in highly exposed individuals (e.g., miners and mercury exposure, people living in areas with substantial environmental contamination) (Bernhoft 2012; Lubick 2010; Nyland et al. 2011). In contrast, our study was based on a representative sample of the U.S. population wherein most participants presumably had only background exposures to xenobiotics. Thus, given certain limitations, we cautiously interpret our findings as somewhat reassuring from a public health perspective because there were very few statistically significant associations between xenobiotic concentrations and ANA. However, the triclosan results raise questions that will require further study.

A major limitation of our study was the assessment of exposure at a single time point. Although a single measure of serum TCDD should be reasonably reflective of body burden or long-term exposure, owing to its half-life of approximately a decade in adults (Wolfe et al. 1994), spot urine concentrations of some nonpersistent compounds are unlikely to provide good representations of long-term, average exposure. For instance, multiple spot urine specimens taken from women during pregnancy typically demonstrate low reproducibility for exposure to BPA (Jusko et al. 2014) and organophosphate pesticide metabolites (Spaan et al. 2015). Consequently, some chemicals may be more susceptible to exposure misclassification than others, and this misclassification is largely dependent on their persistence in the matrices used to assess exposure. In addition to problems with variability, temporality is an issue for chemicals with short half-lives because ANA positivity would have developed before biospecimen collection, and we had no historical exposure information. Prospective cohort studies with measurements taken over time would be required to investigate causality (e.g., for triclosan).

Another limitation was that whereas some chemical concentrations were determined in each 2-year cycle, others were determined in only one or two of the three NHANES cycles, which reduced our statistical power to detect associations between ANA and chemical concentrations. More substantially, some chemicals of interest could not be evaluated because the CDC chemical analysis subsample did not overlap with our ANA subsample. Examples include perfluorinated alkyl substances and phthalates, both of which may exert immunotoxic effects (DeWitt et al. 2012; Grandjean et al. 2012; Hoppin et al. 2013).

A potential limitation of the present study concerns possible model misspecification with regard to confounders. The inclusion or exclusion of true confounders may have over- or underestimated our ANA associations with exposure. For example, a previous analysis of NHANES data suggested an association between ANA and a mixture of dioxin-like PCBs in females (Gallagher et al. 2013). In that analysis, all nondetects were replaced by LOD divided by the square root of 2, and two of the three NHANES cycles were ignored because the proportion of nondetects was extremely large. By comparison, our analysis did not find this association to be statistically significant (*p* < 0.05) despite our using data from all three cycles and reducing bias by treating nondetects as censored. Further investigation revealed that the significance that was originally reported depended mainly on excluding age as a predictor; see the last two columns in Table 2 of Gallagher et al. (2013), which correspond to including and excluding age, respectively. Using the data and covariates of Gallagher et al (2013), neither our lognormal analysis nor their logistic analysis showed a significant association between ANA and the PCB mixture when age was included in the model, but both analyses did show an association when age was removed from the model. For example, the lognormal analysis estimated the MCR as 1.05 (95% CI: 0.98, 1.14) when age was included and 1.17 (95% CI: 1.06, 1.29) when age was excluded. However, we believe it is important to adjust for age, particularly because age is related to both ANA and many chemical concentrations. Thus, in a similar vein, although we stratified on sex and parity, and although our regressions included age and demographic factors related to propensity to exposure, there may be important determinants of exposure and ANA that were not included in our models. For consistency and for screening purposes, we included the same covariates for every chemical, but more individualized analyses with different adjustments might reveal new insights in some cases.

Another recent analysis of NHANES data suggested that ANA were associated with total blood mercury but not with urinary mercury (Somers et al. 2015). That analysis used a weighted logistic model for ANA status and a categorical predictor for mercury after substituting LOD divided by the square root of 2 for concentrations below the LOD, but it did not examine inorganic blood mercury because of heavy censoring. Our unweighted lognormal model for mercury, with ANA as a predictor, did not find a significant association for ANA with total blood mercury, inorganic blood mercury, or urinary mercury. Although the two analyses used different models, covariates, and censoring adjustments, closer inspection suggests that the significance of the association between total blood mercury and ANA may have been caused by treating the mercury variable as categorical rather than as quantitative. The main analysis by Somers et al. (2015) created four categories of total blood mercury. Relative to the first category, their 95% CIs for the ANA odds ratio under several covariate-adjusted models did not include 1 for the second and fourth categories but did for the third category, suggesting a possible relationship between ANA and total blood mercury. However, when we fitted the same covariate-adjusted logistic model, except with mercury (or log mercury) as a linear (continuous) predictor rather than as a categorical predictor, its association with ANA was not significant. In general, we recommend using our censored-data approach if a large proportion of concentrations are below the LOD; otherwise, depending on modeling preferences, using the conventional logistic analysis might be preferable if censoring is limited.

An additional concern is that our mixture analyses assumed that TEFs, which are based primarily on *in vivo* exposures in rodents, apply to assessments of the immune system in humans. TEFs are single-point potency estimates developed from an evaluation of a range of potencies for a given chemical inducing different end points. As such, TEFs may under- or overestimate the actual potency of a chemical for certain end points (Frawley et al. 2014; Trnovec et al. 2013; Van den Berg et al. 2006). To the extent that the TEF for a given chemical may differ from its actual potency for immune effects in humans, some distortion may be introduced into the mixtures analyses. In a conventional analysis, underestimating TEFs in a logistic model for ANA positivity should bias the estimated TEQ regression coefficient upward but should not affect power. However, the lognormal TEQ model focuses on the ratio of mean mixture concentrations for ANA-positive versus ANA-negative participants; thus, the estimated ANA regression coefficient should not be biased if all TEFs are underestimated by a fixed proportion because the constant bias factor would cancel out in both the numerator and the denominator of the ratio. Errors are unlikely to act as a simple scale change, however, and the estimated association between ANA and the TEQ would likely be biased toward the null. Nevertheless, although TEFs may represent an oversimplification, they provide a first approximation for an exploratory analysis of mixture data. TEFs were developed by the WHO and have been used worldwide as the *de facto* method for assessing cumulative exposures to mixtures of dioxin-like compounds and as a means of operationalizing exposure to dioxin-like compounds in human exposure–response relationships (Gallagher et al. 2013).

The present study also had several notable strengths. Environmental exposures were objectively measured (i.e., in serum, whole blood, or urine samples) instead of being assessed via self-reporting in surveys (e.g., fish consumption), and ANA were reliably determined. Unlike analyses that substitute specific values (e.g., LOD/2 or LOD divided by the square root of 2) for nondetects, our analyses did not assume that unknown values were known, thereby avoiding the biases and underestimates of variability that are common in conventional analyses. Furthermore, in contrast to approaches that discard nondetects or analyze detect/nondetect dichotomies, our method allowed full use of the available chemical concentration data. Although regression methods for left- (or right-) censored data have been used previously for individual chemicals with nondetectable concentrations (Dinse et al. 2014), and TEFs have been used to combine detectable concentrations into a mixture TEQ (Van den Berg et al. 2006), our formation of censoring intervals for the TEQ when some component concentrations are below the LOD is a new approach for handling mixtures of congeners.

Detection limits changed both across batches of some assays and over time.Although such changes could be problematic, they are of no consequence for our method if censoring is statistically noninformative about unknown concentrations. This assumption requires that simply knowing the true concentration is below the LOD provides no additional information about the magnitude of the unobserved concentration beyond the fact that it is between zero and the LOD. Noninformative censoring is plausible in the current setting, in which the LOD is primarily a function of the assay properties. Provided that the actual LOD is used for the assay that was applied, the analysis should be valid. Some LODs were found to be systematically lower in recent studies presumably because assay technologies had improved. As a consequence, conventional analyses that focus on the proportion of concentrations above the LOD or that impute using LOD divided by the square root of 2 or LOD/2 could be extremely unreliable, but our censored-data approach avoids this problem.

We performed several sensitivity analyses to validate various aspects of our approach. To evaluate robustness to the choice of concentration distribution, we analyzed the data using several other distributions, including exponential, Weibull, gamma, and log-logistic; all yielded results similar to those obtained from the lognormal distribution that we used (data not shown). We also applied the reverse-scale Cox method (Dinse et al. 2014) to the data for individual chemicals, and the results were not materially different (data not shown). With respect to reversing the roles of outcome and exposure, we refer readers to the simulations reported by Dinse et al. (2014), which showed that outcome/exposure reversal produced valid results over a range of circumstances. Finally, these same simulations showed that valid results were obtained when the proportion of concentrations below the LOD was as high as 90%, which is the value that we used as our highest permitted fraction censored in the present analysis.

## Conclusions

This investigation of xenobiotics and ANA in a nationally representative sample of the U.S. population suggests that background levels of most of the environmental chemicals assessed, with the notable exception of triclosan in males, are not strongly associated with ANA. Future studies should ideally reduce exposure misclassification by including prospective measurement of the chemicals of concern and should track changes in ANA and other autoantibodies over time.

## Supplemental Material

(600 KB) PDF
