# Supplementary material for: Associations Between Selected Xenobiotics and Antinuclear Antibodies in the National Health and Nutrition Examination Survey, 1999–2004
Source: Environ Health Perspect. 2015 Aug 7;124(4):426–36. doi: 10.1289/ehp.1409345 (PMC4829978; doi:10.1289/ehp.1409345)
Supplement: (600 KB) PDF [file ehp.1409345.s001.acco.pdf]

Environ Health Perspect
DOI: 10.1289/ehp.1409345

Note to Readers: *EHP* strives to ensure that all journal content is accessible to all readers. However, some figures and Supplemental Material published in *EHP* articles may not conform to 508 standards due to the complexity of the information being presented. If you need assistance accessing journal content, please contact ehp508@niehs.nih.gov. Our staff will work with you to assess and meet your accessibility needs within 3 working days.

Supplemental Material

Associations between Selected Xenobiotics and Antinuclear Antibodies in the National Health and Nutrition Examination Survey, 1999-2004

Gregg E. Dinse, Todd A. Jusko, Irene Z. Whitt, Caroll A. Co, Christine G. Parks, Minoru Satoh, Edward K.L. Chan, Kathryn M. Rose, Nigel J. Walker, Linda S. Birnbaum, Darryl C. Zeldin, Clarice R. Weinberg, and Frederick W. Miller

Table of Contents

Statistical Model
Accounting for censoring
Accounting for sampling
References
Table S1. Descriptive summaries and ANA associations in selected subgroups of 4,340 participants studied in the 1999-2004 National Health and Nutrition Examination Surveys (NHANES).
Figure S1. Distribution of triclosan concentration by sex, parity, and ANA status, National Health and Nutrition Examination Survey, 1999-2004. Nonparametric Kaplan-Meier curves (Kaplan and Meier 1958) for ANA-positive (solid line) and ANA-negative (dotted line) participants are plotted separately for (A) males, (B) females, (C) nulliparous females, and (D) parous females. For each (x,y) point along a curve, the y-value is the estimated proportion of

participants with measurements below the triclosan concentration represented by the x-value. Concentrations below the limit of detection were treated as left censored when constructing the curves, which were not adjusted for covariates (age, race/ethnicity, time period, BMI, and PIR).

Statistical Model

Let C denote concentration. We assume C is lognormally distributed, which is equivalent to the natural logarithm of C being normally distributed. Assigning parameters, if *ln*(C) is normally distributed with mean μ and standard deviation σ, then C is lognormally distributed with scale and shape parameters $\theta = exp(\mu)$ and σ, respectively. The median of C is θ, its mean is $\theta \times exp(\sigma^2/2)$, its variance is $\theta^2 \times exp(\sigma^2) \times [exp(\sigma^2) - 1]$, and its probability density function is:

$$f(c) = \frac{1}{c\sigma\sqrt{2\pi}} exp\left\{-\frac{[\ln(c)-\ln(\theta)]^2}{2\sigma^2}\right\}. \quad [1]$$

We use regression modeling to adjust for confounders. Let **Z** be a vector of covariates, such as ANA status and the independent variables listed in Table S1. We make the conventional assumption that σ is constant and μ is a linear function of the covariates, $\mu = \boldsymbol{\beta}\mathbf{z}$, in which case the median of C is $\theta(\mathbf{z}) = exp(\boldsymbol{\beta}\mathbf{z})$, where $\boldsymbol{\beta}$ is a vector of regression coefficients and **z** is a vector of observed covariate values. As the lognormal distribution belongs to the family of accelerated failure time models, each covariate has a multiplicative effect on concentration. That is, if C_0 is the baseline concentration (at $\mathbf{Z} = \mathbf{0}$) and C is some general concentration (at $\mathbf{Z} = \mathbf{z}$), then C has the same distribution as $\theta(\mathbf{z}) \times C_0$.

As an illustration, suppose Z is a single binary (0,1) covariate. If $\beta = ln(2)$, for example, then $\theta(0) = exp(\beta \times 0) = 1$ and $\theta(1) = exp(\beta \times 1) = 2$. Thus, under the assumed lognormal distribution, the median (and mean) concentration for those with Z = 1 is twice as large as for those with Z = 0. Similarly, a 1-unit increase in a quantitative covariate with $\beta = ln(2)$ also corresponds to a doubling in median (and mean) concentration. The same interpretation holds when **Z** is a vector of covariates if we focus on the effect of a single component of **Z** for fixed

values of the other covariates.

We use the LIFEREG procedure in SAS (version 9.3, SAS Institute) to obtain maximum likelihood estimates (MLEs) of σ and $\boldsymbol{\beta}$ for each chemical.

Accounting for censoring

For a given chemical, a detectable concentration produces an uncensored observation, {C = c}, which contributes *f*(c) to the likelihood. Otherwise, a nondetectable concentration, known only to be below the LOD, produces a left-censored observation, {C < LOD}, which contributes *F*(LOD) to the likelihood, where

$$F(LOD) = \int_0^{LOD} f(c)dc \,. \qquad [2]$$

The full likelihood is proportional to a product of terms of the form *f*(c) and *F*(LOD) over all persons, and the MLEs of σ and $\boldsymbol{\beta}$ are the values that maximize the full likelihood.

Now suppose we want to analyze a mixture of dioxin-like chemicals, each of which has a TEF that relates its potency to that of the reference chemical (2,3,7,8-TCDD). We consider the dioxin-like chemicals listed in Table 1, with TEFs that decrease from 1.0 (most potent) to 0.00003 (least potent). The TEF is used as an adjustment factor to transform the concentration of a dioxin-like chemical to the same potency scale as the reference chemical. For example, PCB126 is considered one-tenth as potent as 2,3,7,8-TCDD, and their TEFs are 0.1 and 1.0, respectively; thus, we treat concentration C of PCB126 as toxicologically equivalent to concentration 0.1 × C of 2,3,7,8-TCDD, by multiplying its measured concentration by 0.1 prior to combining with other chemicals in the mixture.

Once the concentrations of dioxin-like chemicals have been expressed in equal potency

units, they can be summed to create a TEQ concentration for the mixture. Consider a person exposed to a mixture of M chemicals. For the i-th chemical (i=1,…,M), let C_i denote its concentration, LOD_i its limit of detection, and TEF_i its toxic equivalency factor. If each concentration can be measured, that person's TEQ for the mixture is the sum of M products of the form $TEF_i \times C_i$. However, if at least one concentration is below its LOD, we must account for censoring. Define an interval $[L_i, R_i]$ that contains the i-th chemical's concentration, where L_i is the left endpoint and R_i is the right endpoint. If C_i is uncensored and equals c_i, then $L_i = R_i = c_i$, whereas if C_i is only known to be below LOD_i, then $L_i = 0$ and $R_i = LOD_i$ (i=1,…,M). Each person's TEQ is viewed as interval censored, where the left and right endpoints of the censoring interval are L and R, which equal the sums of $TEF_i \times L_i$ and $TEF_i \times R_i$, respectively, over the M chemicals. Intuitively, the smallest the sum could be is L and the largest the sum could be is R.

For example, consider a binary mixture (M = 2). If the first chemical is detectable with measured concentration c_1 (uncensored) and the second chemical is undetectable with limit of detection LOD_2 (left censored), then the TEQ for the binary mixture is known to be between a lower bound $TEF_1*c_1 + TEF_2*0$ and an upper bound $TEF_1*c_1 + TEF_2*LOD_2$ (interval censored). That is, the TEQ is known to fall in the interval $[TEF_1*c_1, TEF_1*c_1 + TEF_2*LOD_2]$.

As with the individual chemicals, we use the LIFEREG procedure in SAS to fit a lognormal model to interval-censored TEQ data for the mixture being investigated. For each person, we observe $\{L \leq TEQ \leq R\}$. The corresponding likelihood contribution is $F(R) - F(L)$, except in the rare situation of no censoring, in which case L = R and the contribution is $f(L)$, or equivalently $f(R)$. The full likelihood is proportional to a product of such terms over all persons and, as before, the MLEs of σ and $\boldsymbol{\beta}$ are the values that maximize the full likelihood.

Note that because the sum of lognormal variables is not itself lognormally distributed, the concentrations of individual chemicals and their mixtures cannot both be truly lognormally distributed. In practice, however, statistical models are simply approximations to reality. Hence, while the lognormal assumption (or virtually any parametric assumption) cannot be strictly true for both a TEQ concentration and its component concentrations, we apply the same analysis in both cases to be consistent and as a reasonable method of adjusting for confounders and heavy censoring when assessing the association between a mixture concentration and ANA positivity.

For a given mixture, rather than a concentration being left censored by the LOD, suppose a person had no information for a component chemical. In this case, the TEQ censoring interval for the mixture cannot be calculated and one might elect to simply exclude that person from the analysis. To accommodate missing data more efficiently, we instead treat measurement C_i as censored in the interval $[0, MAX_i]$ for anyone with no information on the i-th chemical, where MAX_i is the largest observed concentration for the i-th chemical ($i=1,\ldots,M$) across all persons.

Accounting for sampling

In order to account for the complex NHANES sampling design, we use jackknife methods to obtain appropriate variance estimates for the MLEs (SAS 2011), whether the outcome is an individual chemical concentration or a mixture TEQ concentration. The jackknife approach creates K replicate data sets, where K equals the number of primary sampling units (PSUs). Each replicate data set excludes a different PSU; an MLE is calculated from the remaining data; and the variance of the full-data MLE is estimated by a weighted sum of squared deviations between the replicate-specific MLEs and the full-data MLE.

The 1999-2004 NHANES data involves K = 87 clusters (PSUs) and 43 strata. One

stratum contains three clusters and each of the other 42 strata contains two clusters. Without loss of generality, suppose the first stratum is the one with three clusters and let the j-th replicate data set be the one that excludes the j-th cluster (j=1,…,K). Define a set of jackknife coefficients $\{h_j\}$, where $h_1 = h_2 = h_3 = 2/3$ and $h_j = ½$ for j=4,…,87. For the j-th replicate data set, all observations receive a replicate weight of 1.0, except for those in the stratum with the excluded PSU, where observations in the j-th cluster receive a replicate weight of $1/h_j$ (*i.e.*, either 3/2 or 2). Let γ denote the parameter of interest, which in our lognormal analysis could be either σ or an element of the vector $\boldsymbol{\beta}$. The jackknife estimate of variance for $\hat{\gamma}$, the full-data MLE of γ, is:

$$\hat{V}(\hat{\gamma}) = \sum_{j=1}^{K} h_j \left(\hat{\gamma}_j - \hat{\gamma}\right)^2 , \qquad [3]$$

where $\hat{\gamma}_j$ is the MLE of γ from the j-th replicate data set based on using the replicate weights. We calculate the left and right endpoints of a 95% confidence interval (CI) for γ using the formula:

$$\hat{\gamma} \pm 1.96 \times \sqrt{\hat{V}(\hat{\gamma})} , \qquad [4]$$

where 1.96 is the 97.5-th percentile of the standard normal distribution. The endpoints of a CI for a component of $\boldsymbol{\beta}$, say β_1, can be exponentiated to produce a CI for $exp(\beta_1)$, which is the mean concentration ratio (MCR) when β_1 is the regression coefficient for ANA.

References

Kaplan EL, Meier P. 1958. Nonparametric estimation from incomplete observations. J Amer Statist Assoc 53:457-481.

Kuczmarski RJ, Ogden CL, Guo SS, Grummer-Strawn LM, Flegal KM, Mei Z, et al. 2002. 2000 CDC Growth Charts for the United States: methods and development. Vital Health Stat

Series 11, Number 246:1-190.

SAS. 2011. Jackknife Method. SAS/STAT 9.3 User's Guide. SAS Institute Inc., Cary, NC. http://support.sas.com/documentation/cdl/en/statug/63962/HTML/default/viewer.htm#statug_surveymeans_a0000000239.htm [accessed 27 July 2015].

Table S1. Descriptive summaries and ANA associations in selected subgroups of 4,340 participants studied in the 1999-2004 National Health and Nutrition Examination Surveys (NHANES).

Factor	Number (%) in Category	Percent ANA Positive	Odds Ratio for ANA Positivity [a]
Sex/Parity			
Male	2,199 (51%)	11	Reference
Nulliparous Female	733 (17%)	14	1.3 (1.0, 1.7)
Parous Female	1,257 (29%)	20	1.9 (1.5, 2.3)
Missing (female, parity unknown)	151 (3%)	24	-----
Race/Ethnicity			
Non-Hispanic White	2,018 (47%)	14	Reference
Non-Hispanic Black	954 (22%)	16	1.3 (1.0, 1.6)
Hispanic	1,368 (32%)	14	1.1 (0.8, 1.3)
Time Period (NHANES Cycle)			
1999–2000	1,565 (36%)	12	Reference
2001–2002	1,092 (25%)	18	1.7 (1.3, 2.2)
2003–2004	1,683 (39%)	14	1.2 (1.0, 1.5)
Age (years)			
12–19	1,107 (26%)	12	1.0 (0.7, 1.3)
20–54	1,899 (44%)	14	Reference
55+	1,334 (31%)	16	1.1 (0.9, 1.4)
Mean ± SD [b]	41.6 ± 22.3	-----	-----
Poverty Index Ratio (PIR)			
Below Poverty (< 1)	888 (20%)	13	0.9 (0.7, 1.2)
At or Above Poverty (≥ 1)	3,080 (71%)	14	Reference
Missing	372 (9%)	17	-----
Mean ± SD [b]	2.4 ± 1.6	-----	-----
Body Mass Index (BMI, kg/m^2) [c]			
Underweight	70 (2%)	19	1.2 (0.6, 2.3)
Normal	1,590 (37%)	16	Reference
Overweight	1,319 (30%)	13	0.7 (0.6, 0.9)
Obese	1,248 (29%)	14	0.7 (0.6, 0.9)
Missing	113 (3%)	17	-----

Abbreviations: ANA, antinuclear antibodies; CDC, Centers for Disease Control and Prevention.

[a] Point estimates and 95% confidence intervals under a logistic regression model that adjusts for the other factors in the table and includes only the 3,754 participants with no missing values.

[b] Unadjusted mean and standard deviation of quantitative factors, after excluding missing values.

[c] BMI was classified respectively as underweight, normal, overweight, or obese using standard cut points of <18.5, 18.5 to <25, 25 to <30, or ≥30 in adults (age 20+) and using 2000 CDC growth chart percentiles of <5, 5 to <85, 85 to <95, or ≥95 (adjusted for sex, age, weight, height, and head circumference) in adolescents (age <20) (Kuczmarski et al. 2002).

DOI: 10.1289/ehp.1409345

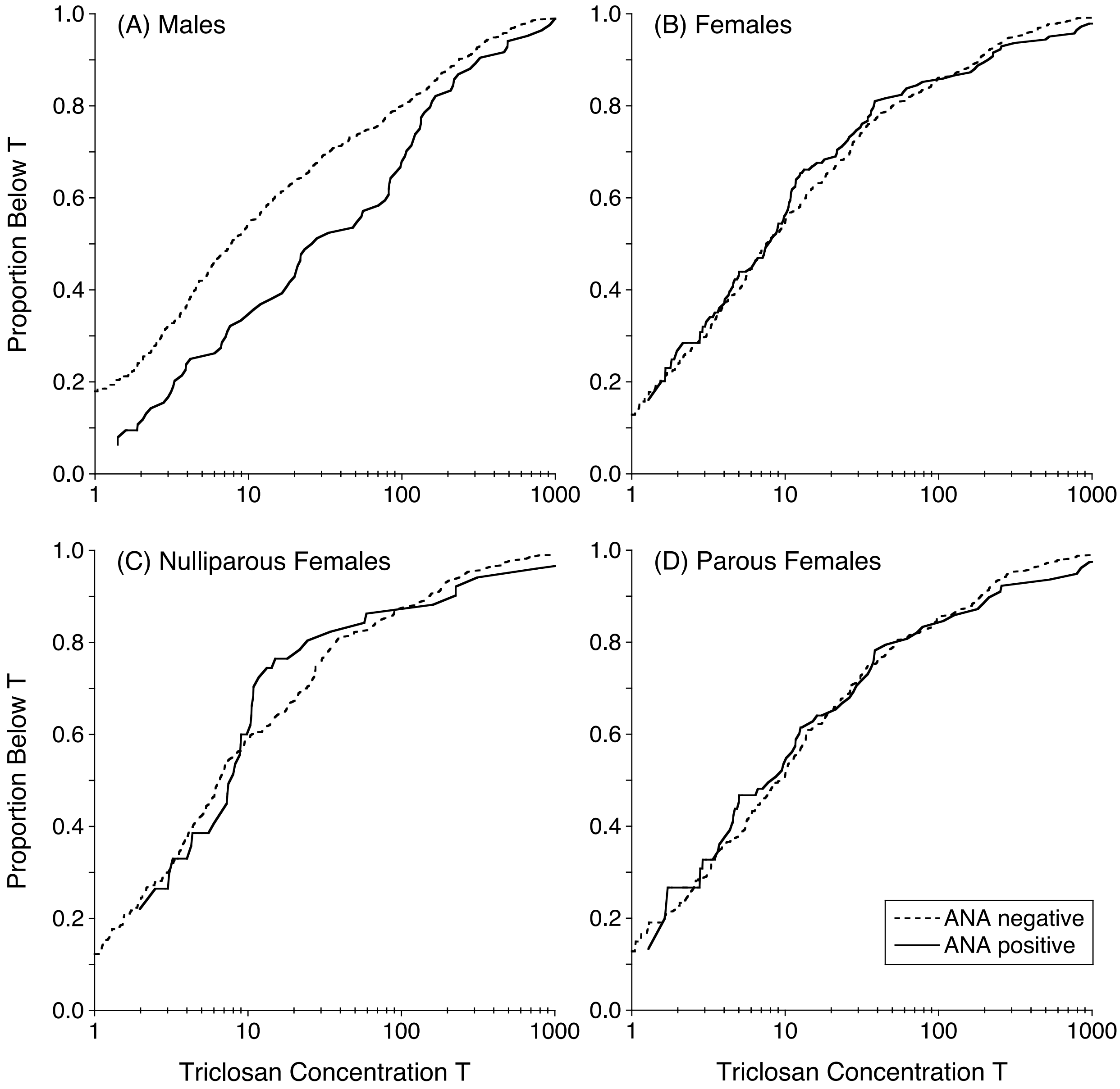

Figure S1. Distribution of triclosan concentration by sex, parity, and ANA status, National Health and Nutrition Examination Survey, 1999-2004. Nonparametric Kaplan-Meier curves (Kaplan and Meier 1958) for ANA-positive (solid line) and ANA-negative (dotted line) participants are plotted separately for (A) males, (B) females, (C) nulliparous females, and (D) parous females. For each (x,y) point along a curve, the y-value is the estimated proportion of participants with measurements below the triclosan concentration represented by the x-value.

Concentrations below the limit of detection were treated as left censored when constructing the curves, which were not adjusted for covariates (age, race/ethnicity, time period, BMI, and PIR).
